# Dopamine and Serotonin-Induced Modulation of GABAergic and Glutamatergic Transmission in the Striatum and Basal Forebrain

**DOI:** 10.3389/fnana.2017.00042

**Published:** 2017-05-11

**Authors:** Toshihiko Momiyama, Takuma Nishijo

**Affiliations:** Department of Pharmacology, Jikei University School of MedicineTokyo, Japan

**Keywords:** striatum, basal forebrain, synapse, GABA, glutamate

## Abstract

Catecholamine receptor-mediated modulation of glutamatergic or GABAergic transmission in the striatum as well as basal forebrain (BF) has been intensively studied during these two decades. In the striatum, activation of dopamine (DA) D2 receptors in GABAergic terminals inhibits GABA release onto cholinergic interneurons by selective blockade of N-type calcium channels. In the BF, glutamatergic transmission onto cholinergic projection neurons is inhibited via DA D1-like receptors by selective blockade of P/Q-type calcium channels. On the other hand, presynaptic inhibition of the GABA release onto cholinergic neurons mediated by D1-like receptors or 5-HT_1B_ receptors is independent of calcium influx. In addition, the DA receptor-mediated calcium influx dependent presynaptic inhibition mentioned above decreases with postnatal development, with selective coupling between DA receptors and each subtype of calcium channels being unchanged. Furthermore, the precise origin of these GABAergic or glutamatergic inputs to postsynaptic neurons can be identified by recent optogenetic approaches. Thus, modulatory mechanisms in specific synaptic connections between certain types of neurons in the striatum and BF are being identified.

## Introduction

Nigro-striatal dopaminergic pathway plays important roles in motor control through the interaction between dopamine (DA) and acetylcholine (ACh; Crag, 2006; Pisani et al., 2007). Previous studies have suggested that disorders of the system could be involved in the basal ganglia-related diseases such as Parkinson’s disease (for review, Kreitzer and Malenka, 2008). On the other hand, basal forebrain (BF) nuclei are the origin of cholinergic neurons which project to various brain regions containing cortex and hippocampus (Rye et al., 1984), and have been shown to be involved in attention, arousal, learning, memory and sleep-wake states, as well as the related disorder, including dementia and Alzheimer’s disease (Coyle et al., 1983; Oyanagi et al., 1989; Zant et al., 2016).

One of the potential neurophysiological events contributing to the striatum- or BF-related control of brain function mentioned above, is synaptic transmission in these brain regions (Calabresi et al., 2007). Presynaptic modulation of excitatory and inhibitory transmitter release in the striatum as well as BF has been intensively studied during these two decades. Especially, recent studies have clarified ionic mechanisms underlying DA- or serotonin receptor-mediated presynaptic modulation of glutamate and GABA release in these forebrain regions; modulation coupled to certain subtypes of calcium channels (Momiyama and Koga, 2001; Momiyama and Fukazawa, 2007) or modulation independent of calcium influx (Momiyama and Sim, 1996; Nishijo and Momiyama, 2016) among different synapses. Unique profiles of postnatal developmental changes in these synaptic modulation have also been clarified (Momiyama, 2003, 2010). Furthermore, specific connections between certain types of striatal neurons have been gradually unveiled by optogenetic approaches applied to these brain regions.

This mini review article focuses on the DA- and serotonin receptor-induced modulation of neurotransmitter release through different types of calcium channels as well as in calcium influx-independent manner, in relation to the involvement of different calcium channels in neurotransmitter release in the striatum and BF.

### Multiple Types of Calcium Channels Mediate Central Synaptic Transmission

Central fast synaptic transmission is regulated by multiple types of Ca^2+^ channels including N-, P/Q-, R- and other unknown types (Takahashi and Momiyama, 1993; Wheeler et al., 1994). Using selective blockers, previous studied have estimated that the coefficient for the functional relationship between presynaptic Ca^2+^ concentration and transmitter release is 3 or 4 (Dodge and Rahamimoff, 1967; Augustine and Charlton, 1986; Takahashi, 1992; Takahashi and Momiyama, 1993; Momiyama and Koga, 2001). On the other hand, a recent study has found that, in the GABAergic synaptic transmission onto BF cholinergic neurons, unlike the previous studies (Dodge and Rahamimoff, 1967; Augustine and Charlton, 1986; Takahashi, 1992; Takahashi and Momiyama, 1993; Momiyama and Koga, 2001), the relation between extracellular calcium concentration and transmitter release follows a power of less than 2 instead of 3–4 (Nishijo and Momiyama, 2016), suggesting variable transmission mechanisms, including release probability, number of release sites and localization of each subtype of Ca^2+^ channels in relation to release sites, among central synapses.

### Coupling between Dopamine D_2_ Receptors and N-Type Calcium Channels

**Figure 1:**
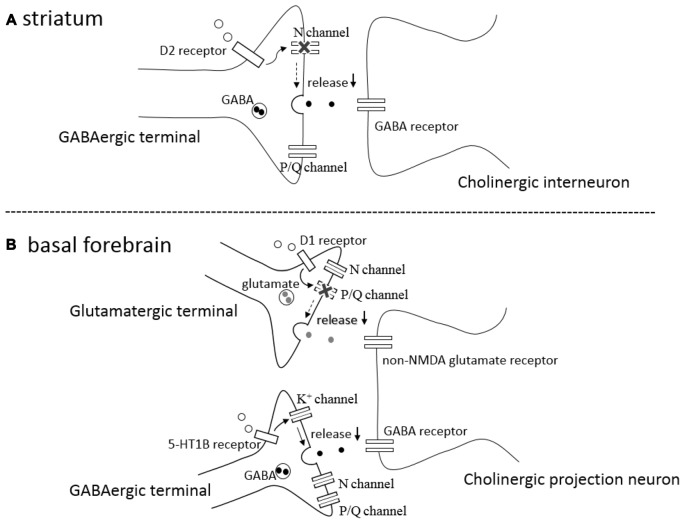
**(A)** A schematic drawing of a GABAergic synapse onto a striatal cholinergic interneuron showing that activation of presynaptic D2-like receptors (D_2_ receptor) selectively blocks N-type calcium channels (N channel) without affecting P/Q-type calcium channels (P/Q channel), leading to inhibition of GABA release (↓). **(B)** A schematic drawing of a glutamatergic and GABAergic synapses onto a cholinergic projection neuron in the basal forebrain (BF), showing that activation of presynaptic D1-like receptors (D_1_ receptor) selectively blocks P/Q-type calcium channels (P/Q channel) without affecting N-type calcium channels (N channel), inhibiting glutamate release (↓), and that activation of presynaptic serotonin 5-HT 1B receptors (5-HT_1B_ receptor) modulates potassium channels (K^+^ channel) affecting neither N- type nor P/Q-type calcium channels, resulting in the reduction (↓) of GABA release.

In the rat striatum, GABAergic synaptic transmission onto cholinergic interneurons is inhibited by activation of presynaptic D2-like DA receptors on the GABAergic terminals (Pisani et al., 2000; Momiyama and Koga, 2001). The inhibitory effects induced by agonist activation of D2-like receptors are occluded after blocking N-type Ca^2+^ channels by ω-conotoxin (ω-CgTX), whereas the inhibition remained unaffected after blocking P/Q-type Ca^2+^ channels by ω-Aga-IV-A (Momiyama and Koga, 2001), suggesting that activation of presynaptic D2-like receptors selectively blocks N-type Ca^2+^ channels, thereby inhibiting GABA release (Figure 1A). The selective coupling is interesting, since the involvement of N-type Ca^2+^ channels is less than that of P/Q-type Ca^2+^ channels in this synapse (Momiyama and Koga, 2001). Among cloned D1–D5 DA receptors, D2-like receptors contains D2, D3 and D4 subtypes. The findings using DA receptor knock-out mice confirmed the involvement of D2 subtype in the D2-like receptor mediated inhibition of GABAergic transmission and selective coupling between D2-like receptors and N-type Ca^2+^ channels (Sato et al., 2014; Yamada et al., 2016).

### Coupling between Dopamine D_1_ Receptors and P/Q-Type Calcium Channels

In the BF, glutamatergic transmission onto cholinergic neurons is inhibited by activation of presynaptic D1-like DA receptors (Momiyama and Fukazawa, 2007). Unlike the case of D2-like receptor-mediated presynaptic inhibition of GABA release in the striatum mentioned above, D1-like receptor-mediated inhibition is occluded after blocking P/Q-type Ca^2+^ channels by ω-Aga-IV-A, whereas the inhibition remained unaffected after blocking N-type Ca^2+^ channels by ω-CgTX (Momiyama and Fukazawa, 2007), suggesting selective coupling between D1-like DA receptors and P/Q-type Ca^2+^ channels in the modulation of glutamate release onto cholinergic neurons in the BF (Figure 1B).

### Postnatal Developmental Change

**Figure 2:**
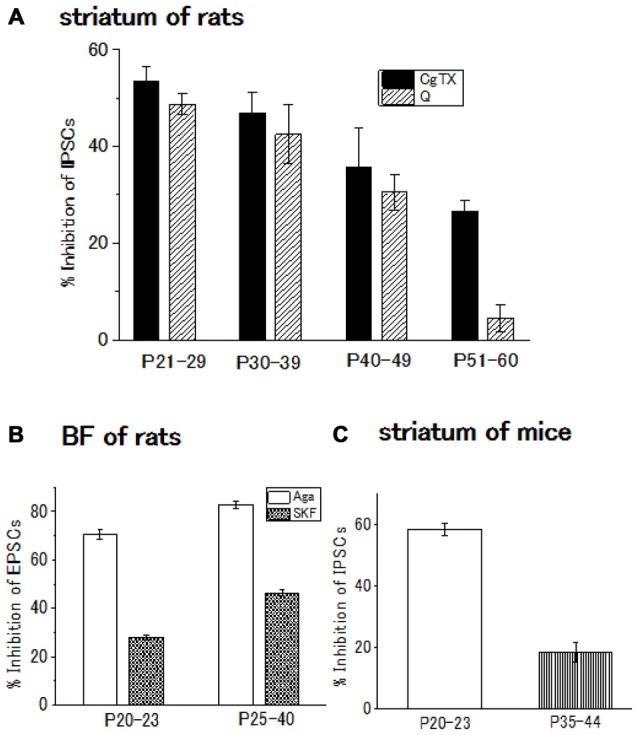
**(A)** Developmental decrease in the inhibitory effect of ω-conotoxin (CgTX), an N-type Ca^2+^ channel blocker, or quinpirole (Q), a dopamine (DA) D2-like receptor agonist, on the GABAergic inhibitory postsynaptic currents (IPSCs) evoked in striatal cholinergic interneurons of rats. Each bar shows the mean with SEM derived from 3 to 16 cells. Reproduced from the values published in Momiyama (2003). **(B)** Developmental increase in the inhibitory effect or SKF81297 (SKF), a D1-like receptor agonist, or ω-agatoxin (Aga), a P/Q-type Ca^2+^ channel blocker Ca^2+^ channel blocker on the glutamatergic excitatory postsynaptic currents (EPSCs) evoked in BF cholinergic neurons of rats. Each bar shows the mean with SEM obtained in 3–36 cells. Reproduced from the values published in Momiyama (2010). **(C)** Developmental decrease in the inhibitory effect of CgTX on the GABAergic IPSCs evoked in striatal cholinergic interneurons of mice. Each bar shows the mean with SEM derived from 6 to 7 cells. Reproduced from the values published in Sato et al. (2014).

The contribution of N-type Ca^2+^ channels declines with development and almost diminishes within postnatal 2 weeks in the Calyx of Held (Iwasaki and Takahashi, 1998), whereas the contribution remained unchanged in the spinal cord (Iwasaki et al., 2000). In the striatum of rats or mice, the postnatal developmental change undergoes an intermediate pattern; in the GABAergic transmission onto striatal cholinergic interneurons, N-type Ca^2+^ channel contribution gradually decline until postnatal 60 days (Momiyama, 2003). In addition, D2-like DA receptor-mediated presynaptic inhibition decreases with age in parallel with the decline in the contribution of N-type Ca^2+^ channels to the synaptic transmission (Momiyama, 2003; Sato et al., 2014; Yamada et al., 2016; Figures 2A,C).

In the BF glutamatergic transmission onto cholinergic neurons of the rat, selective coupling between D1-like receptors and P/Q-type Ca^2+^ channels mentioned above (Momiyama and Fukazawa, 2007) remains with postnatal development. However, in this glutamatergic synapse, both D1-like receptor-mediated presynaptic inhibition of glutamate release and contribution of P/Q-type Ca^2+^ channels to the synaptic transmission increase in parallel (Momiyama, 2010; Figure 2B), while D2-like receptor-mediated presynaptic inhibition and the N-type Ca^2+^ channels contribution to the synaptic transmission both decrease in the striatum during development (Momiyama, 2003; Sato et al., 2014; Figures 2A,C).

### Calcium Influx-Independent Modulation of Transmitter Release

A recent study has demonstrated that GABAergic synaptic transmission onto cholinergic neurons in the BF is inhibited by activation of presynaptic 5-HT_1B_ receptors, and that the inhibition remained unchanged after blocking N-, P/Q-, or R-type Ca^2+^ channels by selective blockers of each Ca^2+^ channel subtype (Nishijo and Momiyama, 2016). Although the modulation might be regulated by other unknown types of Ca^2+^ channels, the finding could suggest that 5-HT_1B_ receptor mediated presynaptic inhibition of GABA release is independent of Ca^2+^ channels and/or Ca^2+^ influx to presynaptic terminals. In addition, 5-HT_1B_ receptor-mediated inhibition was blocked in the presence of 4-AP, a potassium channel blocker, suggesting the involvement of potassium channel modulation in the inhibition of GABA release (Figure 1B). It remains unknown whether Ca^2+^-dependent and Ca^2+^-independent modulation of transmitter release play different roles in these neuronal circuits.

### Implications

Postnatal days 60 is the age between adolescence and young adult in the rat (Kokoshka et al., 2000). Therefore, the developmental change in the contribution of N-type Ca^2+^ channels to striatal synaptic transmission could be involved in the control of vivid motion or complex behaviors prominent especially in younger ages. These findings also indicate that pharmacological manipulation of N-type Ca^2+^ channels could be at least one of the therapeutic tools for the age-dependent treatment of basal ganglia-related diseases.

On the other hand, BF cholinergic system has been implicated in attention, motivation or memory (Arendt et al., 1989; Dunnett and Fibiger, 1993; Muir et al., 1994) as well as in the related disorders such as Alzheimer’s disease (Price et al., 1986; Mann, 1988; Perry et al., 1993; Gaula and Mesulam, 1994). Therefore, the findings regarding the selective coupling between D1-like receptors and P/Q-type Ca^2+^ channels in the modulation of BF glutamatergic transmission suggest possible involvement of P/Q-type Ca^2+^ channels in these neuropsychiatric functions. Furthermore, a recent finding regarding the involvement of potassium channel modulation in the inhibition of GABA release (Nishijo and Momiyama, 2016) suggests some supplementary role of potassium channels in these functions as well as in the related disorders.

### Perspective

In above-mentioned patch-clamp studies using brain slice preparations of rats or mice, the precise origins of GABAergic or glutamatergic synaptic inputs to striatal or BF neurons are unidentified. The limitations have been overcome with recent advance in optogenetic technique, where certain populations of neurons can be activated by optical stimulation of channelrhodopsin-2 expressed in the neurons. Actually, major origins of GABAergic synaptic inputs onto striatal cholinergic interneurons have been identified to be medium spiny neurons within the striatum (Chuhma et al., 2011). Also, functional synaptic inputs from BF cholinergic neurons to basolateral amygdala neurons have been shown (Unal et al., 2015). Using this technique, input-specific modulation of synaptic transmission as well as selective coupling between receptors and channels will be clarified.

It has been shown that P/Q-type Ca^2+^ channels are expressed on the axon terminal of parvalbumin (PV)-containing interneurons in the cortex (Castejon et al., 2016). PV-containing GABAergic neurons are also one of the main neuronal populations in the BF, sending GABAergic inputs to cholinergic neurons (Duque et al., 2000; Zaborszky and Duque, 2000). Therefore, PV-containing GABAergic neurons in the BF might also express a certain subtypes of Ca^2+^ channels, regulating GABA release onto cholinergic neurons. The issues will be also clarified by future studies using optogenetics in combination with ultrastructural technique.

## Author Contributions

TN and TM equally contributed the experiments and data analyses in the studies cited in the review article. TN wrote the first draft of the manuscript, and TM revised it in discussion with TN.

## Funding

This work was supported by a Grant-in-Aid for Scientific Research from the Ministry of Education, Culture, Sports, Science and Technology of Japan (no. 21500374 and 24500464) to TM.

## Conflict of Interest Statement

The authors declare that the research was conducted in the absence of any commercial or financial relationships that could be construed as a potential conflict of interest.
